# Serious Consequences of Perforation of Appendix

**Published:** 1893-02

**Authors:** 


### SERIOUS CONSEQUENCES OF PERFORATION OF APPENDIX.

It has fallen to the lot of the writer, during the past few years of his professional experience, to make post-mortem examinations in four cases of perforation of the veriform appendix, which died without operation, and to excise the appendix in a fifth case, which succumbed before the operation was ended.

From the comparatively small place allowed this grave complication of appendicitis by the writers on this subject, I am impressed with the fact that others have not been called upon to note so frequently this fatal result. It may be supposed that timely surgical interference would have avoided this calamity. But the rapidity of the progress towards collapse was such as to afford little opportunity for relief after making a diagnosis of perforation. In one of the cases, which was seen in consultation with three other colleagues some hours after the developments pointed to perforation, the collapse was so marked that they overruled the proposition in favor of immediate operation, upon the ground that the patient would be likely to die under the knife. He survived fourteen hours without reaction, and as there could be no reasonable expectation of relief, except by an operation, it would have been legitimate surgery to have given him this chance for escape from certain death. All the concomitants of perforation of the appendix indicate a speedy fatal termination, not from the comparatively slow process of peritonitis, but from the overpowering depression of the vital forces, partaking of the nature of shock. The cause must, therefore, be removed with the least possible delay, by cutting down upon the opening and excising the diseased part, with suture of the proximal portion of the appendix.

If the procedure did not go beyond these steps, little would be gained, as the fecal exudation with its toxic properties has penetrated the abdominal cavity, and a septic process of the most virulent nature has been propagated to the peritoneum. A veritable necrosis of the tissues seems to result from the contact of fecal matter under such circumstances, and it is not only requisite to wash out every particle of the exudation, but to apply correctives to the surfaces involved by the contamination.

In determining upon a proper antiseptic for the arrest of the disintegrating process in the peritoneum, carbolic acid and sublimate solutions should be excluded and the preference given to washes of chloride of sodium, boracic acid and thymol. A drainage tube, extending from the affected part, should be secured in the external wound for irrigation afterwards.

This notice of a special feature of trouble connected with the appendix vermiformis, is not given from indifference to the many cases of disturbance in this region which call for prompt attention. It is, on the contrary, intended to accentuate the importance of early action to prevent this grave result of appendicitis. If an exploratory operation should be undertaken in every instance of localized inflammation in the right iliac fossa, without temporizing with medical treatment, it would prove the most effectual security against having to deal with the consequences of perforation. When degeneration of structure is detected by an incision, whether from a foreign body, or indurated fecal concretions within the appendix, there is urgent demand for excision and suture. If, however, an inflammatory process is found to exist in its walls independent of the contents, safety lies in proceeding at once to the radical measure of removal of the appendix. J. McF. G.

---

THE St. Louis Medical and Surgical Journal announces that it has reached its fiftieth year of publication, and to celebrate the event in a befitting manner, the issues of 1893 will be especially prepared. Beside the publication of papers from a number of eminent gentlemen, a list of whom is given, they will print a photograph and a sketch of the life of each one of the editors of the Journal since its inception.

